# The Role of Feature Sharedness in the Hierarchical Organization of Semantic Knowledge

**DOI:** 10.3233/BEN-2012-129011

**Published:** 2013

**Authors:** J. Frederico Marques

**Affiliations:** Faculty of PsychologyUniversity of LisbonLisbonPortugal

**Keywords:** Semantic memory, feature sharedness, superordinate concepts, semantic dementia, stroke aphasia

## Abstract

Data from neuropsychological research suggest that categorizing objects at different levels of specificity requires different cognitive and neural processes. This short paper presents and discusses a theoretical hypothesis for this organization in terms of feature sharedness. It is proposed that superordinate concepts involve a larger absolute number of exemplars that share a particular feature, thus making them more resistant to damage than basic level concepts (i.e. superordinate advantage). Simultaneously, in relative terms, features are less shared overall by superordinate members than by basic level members, which imply higher executive requirements and can conversely lead to superordinate deficits. This hypothesis is discussed in relation to behavioral data from semantic dementia and stroke aphasia patients and fMRI data from healthy subjects that support the role of feature sharedness in the hierarchical organization of semantic knowledge.

## 1. Introduction

When we talk about things, people or objects we can use different names and very often these names and concepts seem to be organized hierarchically, from subordinate (e.g. Dalmatian) to basic level (e.g. Dog) to superordinate concepts (e.g. Animal). Data from neuropsychological research suggest that categorizing objects at different levels of specificity requires different cognitive and neural processes. In this short paper we present and discuss a theoretical hypothesis for this organization in terms of feature sharedness [5,6].

## 2. Hierarchical organization and neuropsychological data

The degradation of semantic knowledge in dementia patients seems to progress from the loss of more specific information to more general and broad concepts [2, 9]. Warrington [9] was the first to systematically report this data pattern, showing a relative sparing of superordinate information (e.g. animal) as compared with specific feature knowledge (e.g. size, colour), and suggesting that this progression reflects the fact that conceptual knowledge is hierarchically organized. In contrast, other semantic impairments, such as some cases of stroke aphasia associated to prefrontal and temporoparietal lesions, albeit less frequently, show better performance at basic than superordinate level processing [1,3].

It has been proposed that the first cases reflect the fact that features of superordinate concepts are more resistant to damage because they are shared by more exemplars than features at lower levels [8]. In contrast, in the latter cases, superordinate impairment suggests that more general classification of objects requires greater executive processes since information must be drawn together from a more disparate set of exemplars in comparison with basic-level categorization [3]. I have proposed that feature sharedness may underlie both these effects [5,6] and allow their understanding within a common framework.

ISSN 0953-4180/13/$27.50 © 2013 – IOS Press and the authors. All rights reserved

## 3. Hierarchical organization and feature sharedness

Feature sharedness corresponds to the extent to which a certain feature is common to all members of a concept. This dimension would explain the superordinate advantage in semantic dementia since superordinate concepts draw together a larger absolute number of exemplars that share a particular feature, thus making these concepts more resistant to damage. At the same time, in relative terms, features are less shared overall by superordinate members, which in turn implies higher executive requirements and explain better performance at basic than superordinate level processing in stroke aphasia.

## 4. Testing the role of feature sharedness

We directly tested the role of feature sharedness by orthogonally manipulating this dimension (more vs. less shared) and concept level (superordinate vs. basic) in a sentence verification task (e.g. "the plant needs water", "the piano is an antique") and considering both reaction times and correct responding. Feature sharedness was manipulated considering previous ratings on this dimension obtained for both superordinate and basic level concepts (from [5]), while controlling for a number of variables that influence performance on this task (concepts matched for linguistic frequency across levels; features were matched for production frequency, distinctiveness and proportion of non-sensory attributes; and sentences matched in length).

## 5. Empirical support for feature sharedness

In a first study (Marques & Charnallet, unpublished data) we evaluated 5 semantic dementia patients together with 10 matched controls (sex, age, education level) in terms of correct performance. Crucial results to the role of feature sharedness showed that independently of concept level, feature sharedness impacts on patients' correct performance while having no effect on the control group, thus supporting the idea that feature sharedness may underlie the superordinate advantage found in these patients.

In a second study (Marques et al., unpublished data) we tested 6 stroke aphasia patients (similarly to [1,3]) that also presented executive deficits (as assessed by performance on the trail-making test and/or the Wisconsin card sort test), together with 12 matched controls (sex, age, education level). While there were no significant differences in terms of correct responding between patients and controls, there were important differences in the time taken to respond. In particular, a significant feature by group interaction showed that patients but not controls were slower to verify sentences about less shared features in comparison with sentences involving more shared features. A significant group by concept level was also observed showing that patients but not controls were slower to verify sentences about superordinates in comparison with sentences about basic level concepts, thus supporting the idea that less feature sharedness and processing of superordinate concepts in general may be associated to higher executive demands.

We have also obtained data in accord with this idea in an fMRI study with 17 healthy participants performing this same feature verification task while in the scanner [7]. Crucially, fMRI results showed that sentences involving less shared features recruited left lateral prefrontal cortex (PFC), suggesting that processing less shared features requires a more controlled semantic retrieval; and in a similar manner sentences involving superordinate concepts implicated left lateral PFC relative to sentences involving basic concepts, suggesting that processing superordinate concepts requires extra executive control. Moreover, a concept by feature interaction was also significant indicating that the left lateral PFC was recruited for all sentences that involved either a superordinate concept or a feature that was less shared by the concept.

## 6. Conclusions

We have directly tested the role of feature sharedness in the hierarchical organization of semantic knowledge by orthogonally manipulating feature sharedness and concept level in a sentence verification task in a set of studies using different populations and methodologies. These results support the importance of feature sharedness to explain the hierarchical organization of semantic knowledge, and in particular that, the different structure of superordinate and basic level concepts in terms of feature sharedness results in different patterns of semantic impairment [5–7]. Moreover, at a more general level, these results support a perspective of the organization of semantic knowledge as the outcome of the structure of the semantic representations in memory (in temporal cortex) and executive processes (in prefrontal regions) that help to retrieve information in a directed manner [4].
